# MCPIP1-induced autophagy mediates ischemia/reperfusion injury in endothelial cells via HMGB1 and CaSR

**DOI:** 10.1038/s41598-018-20195-6

**Published:** 2018-01-29

**Authors:** Xiaolong Xie, Tiebing Zhu, Lulu Chen, Shuang Ding, Han Chu, Jing Wang, Honghong Yao, Jie Chao

**Affiliations:** 1 Department of Cardiology, The First Affiliated Hospital of Nanjing Medical University, Nanjing, Jiangsu 210029 China; 2 Department of Physiology, Medical School of Southeast University, Nanjing, Jiangsu 210009 China; 3 Department of Pharmacology, School of Medicine, Southeast University, Nanjing, Jiangsu 210009 China; 4 Key Laboratory of Developmental Genes and Human Disease, Institute of Life Sciences, Southeast University, Nanjing, Jiangsu 210096 China; 5 Department of Respiration, Zhongda Hospital, School of Medicine, Southeast University, Nanjing, Jiangsu 210009 China

## Abstract

Monocyte chemotactic protein-1-induced protein 1 (MCPIP1) plays a important role in ischemia/reperfusion (I/R) injury. Autophagy is involved in activating endothelial cells in response to I/R. However, researchers have not clearly determined whether MCPIP1 mediates I/R injury in endothelial cells via autophagy, and its downstream mechanism remains unclear. Western blotting analyses and immunocytochemistry were applied to detect protein levels were detected in HUVECs. An *in vitro* scratch assay was used to detect cell migration. Cells were transfected with siRNAs to knockdown MCPIP1 and high mobility group box 1 (HMGB1) expression. The pharmacological activator of autophagy rapamycin and the specific calcium-sensing receptor (CaSR) inhibitor NPS-2143 were used to confirm the roles of autophagy and CaSR in I/R injury. I/R induced HMGB1 and CaSR expression, which subsequently upreguated the migration and apoptosis of HUVECs and coincided with the increase of autophagy. HMGB1 was involved in cell migration, whereas CaSR specifically participated in I/R-induced HUVEC apoptosis. Based on these findings, I/R-induced MCPIP1 expression regulates the migration and apoptosis of HUVECs via HMGB1 and CaSR, respectively, suggesting a new therapeutic targetof I/R injury.

## Introduction

Vascular endothelial cell dysfunction plays an crucial role in ischemia/reperfusion (I/R) injury, a common aspect of cardiovascular disease that is characterized by the over-production of inflammatory factors, such as cytokines and chemokines^1,2^. Various biological events, such as autophagy, endoplasmic reticulum stress (ERS) and ubiquitination, are involved in endothelial cell dysfunction. Autophagy plays an important role in the ability of cells to adapt to changing environmental conditions and in cellular remodeling during angiogenesis^3–6^. However, the mechanisms underlying I/R-induced endothelial cell dysfunction that are associated with autophagy remain poorly understood.

Based on accumulating evidence, monocyte chemotactic protein-1 (MCP-1) and its downstream molecule MCP-1-induced protein 1 (MCPIP1) facilitate vascular inflammation and endothelial dysfunction in response to I/R^1,7–10^. For example, MCPIP1 has been shown to induce angiogenesis during placental vasculogenesis, which in turn leads to vascular remodeling^11–13^. Interestingly, recent studies have linked MCP-1/MCPIP1 with autophagy under different conditions that induce cell activation and apoptosis. For instance, MCP-1 and MCPIP1 contribute to cardiomyoblast death in patients with heart failure and are associated with autophagy resulting from ERS^14^. Moreover, MCPIP1-induced autophagy is required for angiogenesis in patients with angiogenesis-related cardiovascular diseases^15^. On the other hand, MCPIP1/p53 expression is induced after SiO2 exposure and promotes macrophage activation and apoptosis, suggesting the presence of a general link between the MCPIP1 signaling pathway and autophagy in different diseases^16,17^.

Autophagy is an important biological event required to maintain cell homeostasis^5,18,19^. However, several stimuli may cause irreversible cell injury and cell death via autophagy, which contribute to some pathologies^20–23^. According to previous data from our lab, I/R induces the expression of inducible nitric oxide synthase (iNOS), which subsequently increases the migration and apoptosis of human umbilical vein endothelial cells (HUVECs) by promoting autophagy^3^. Moreover, some studies have suggested a direct link between MCP-1 and iNOS, which is consistent with our findings in endothelial cells cultured under I/R conditions^24–26^. However, the downstream effects of I/R-induced MCPIP1 expression on cell migration and apoptosis associated with autophagy remain unknown.

The present study aimed to determine the mechanism by which MCPIP1 regulated I/R-induced HUVEC migration and apoptosis and the specific roles of autophagy in these processes. The conclusions of current research may help to understand the mechanisms regulating MCPIP1 expression and its functional relevance to I/R injury, providing insight into potential therapeutic targets for myocardial ischemia.

## Materials and Methods

### Reagents

Fetal bovine serum (FBS), normal goat serum, Dulbecco’s Modified Eagle’s Medium (DMEM; #1200-046), and 10× MEM (11430–030) were all obtained from Life Technologies. Amphotericin B (BP2645) and the supplement GlutaMAX (35050–061) were obtained from Gibco, and Pen/Strep (15140–122) was obtained from Fisher Scientific. Antibodies against the calcium-sensing receptor (CaSR, sc33821, rabbit), MCPIP1 (sc136750, goat) and glyceraldehyde 3-phosphate dehydrogenase (GAPDH; sc32233, mouse) were obtained from Santa Cruz Biotechnology, Inc. The antibody against high mobility group box 1 (HMGB1) protein (ab18256, rabbit) was obtained from Abcam Biotechnology, Inc. Control siRNA (sc-37007), a non-targeting 20–25-nt siRNA designed as a negative control, was obtained from Santa Cruz Biotechnology, Inc. The reagent used for siRNA transfection was purchased from Santa Cruz Biotechnology, Inc.

### Cell culture

HUVECs were purchased from ScienCell^®^ and maintained as previously described^1,3^.

### Cell-based model simulating ischemia/reperfusion injury

The I/R model used here based upon a version of a described method^1,3,27–30^.

### Lentiviral transduction of HUVECs

HUVECs were transduced with the LV-GFP lentivirus (Hanbio, Inc., Shanghai, CN) as previously described^31–34^. Briefly, HUVECs (passage (P) 3–5) were cultured in a 24-well plate (1 × 10^4^ cells/well) in DMEM supplemented with 10% FBS for 48 h. The medium was then replaced with 1 mL of fresh medium and 8 µg/mL of polybrene. 15 μl of lentivirus solution (10^7^ IU/mL) were added to each well, and the 24-well plate was incubated at 37 °C with 5% CO_2_ for 24 h. Following incubation, the medium was replaced with fresh DMEM containing 10% FBS, and the cells were cultured at 37 °C and 5% CO_2_ until they reached >50% confluence. The transduced cells were selected using puromycin by replacing the medium with DMEM containing 10 µg/mL puromycin and 10% FBS and culturing the cells at 37 °C in a 5% CO_2_ atmosphere for 24 h. The cells were subsequently washed twice with fresh DMEM containing 10% FBS. Transduced and pure HUVEC cultures were expanded and stored in liquid nitrogen as previously described^35^.

### *In vitro* scratch assay

Cell migration in a 2D culture system was evaluated using an *in vitro* scratch assay^1^. To evaluate the effect of cell division on migration, mitomycin pretreatment was applied for one hour. As shown in Fig. S1, mitomycin did not alter the effect of I/R on cell migration, which ruled out the effect of cell division on migration.

### Immunoblotting

Immunoblotting was performed as previously described^3^.

### Immunofluorescence staining

Immunofluorescence staining was performed as previously described^35^. HUVECs (2 × 10^5^ cell/well) were seeded onto coverslips placed on 24-well plates. After treatment, the coverslips were rinsed twice with PBS, and the cells were fixed with 4% paraformaldehyde overnight. The fixed cells were incubated with 0.3% Triton X-100 in PBS for 30 min and then rinsed twice with PBS. The cells were blocked with 10% normal goat serum (NGS) in 0.3% Triton X-100 for 2 h at room temperature and then incubated with primary antibodies overnight at 4 °C. After three washes with PBS, the cells were incubated with fluorescently-labeled secondary antibodies (Alex Flour® 488 and 594, 1:250), and the cell nuclei were stained with DAPI (4,′6-diamidino-2-phenylindole) and mounted with ProLong Gold antifade reagent (Life Technologies®). Images were captured under a fluorescence microscope (Olympus IX70, Olympus America, Inc., Center Valley, PA).

### MTT assay

Cell viability was measured using the 3-(4,5-dimethylthiazol-2-yl)-2,5-diphenyl tetrazolium bromide (MTT) method^31^.

### CCK8 assay

Cell viability was measured using the CCK8 assay (Dojindo, Tokyo, Japan) following the manufacturer’s protocol. Briefly, 10 μl of CCK8 was added to each culture well, and then, the cells were incubated at 37 °C for 2 h. The absorbance at 450 nm was measured using a spectrophotometer. Cell viability was expressed as the percentage of viable cells in the experimental group relative to the control group.

### Hoechst staining

Cells were fixed and stained with 5 μM Hoechst 33324 (Invitrogen) for 15 min at room temperature to quantify the number of apoptotic cells^1,36^.

### Statistics

The data are expressed as the means ± SEM. Unpaired numerical data were compared using either an unpaired t-test (two groups) or an ANOVA (more than two groups). Significance was established at p < 0.05.

## Results

### I/R-induced MCPIP1 expression is associated with up-regulated HMGB1 expression in HUVECs

**Figure 1:**
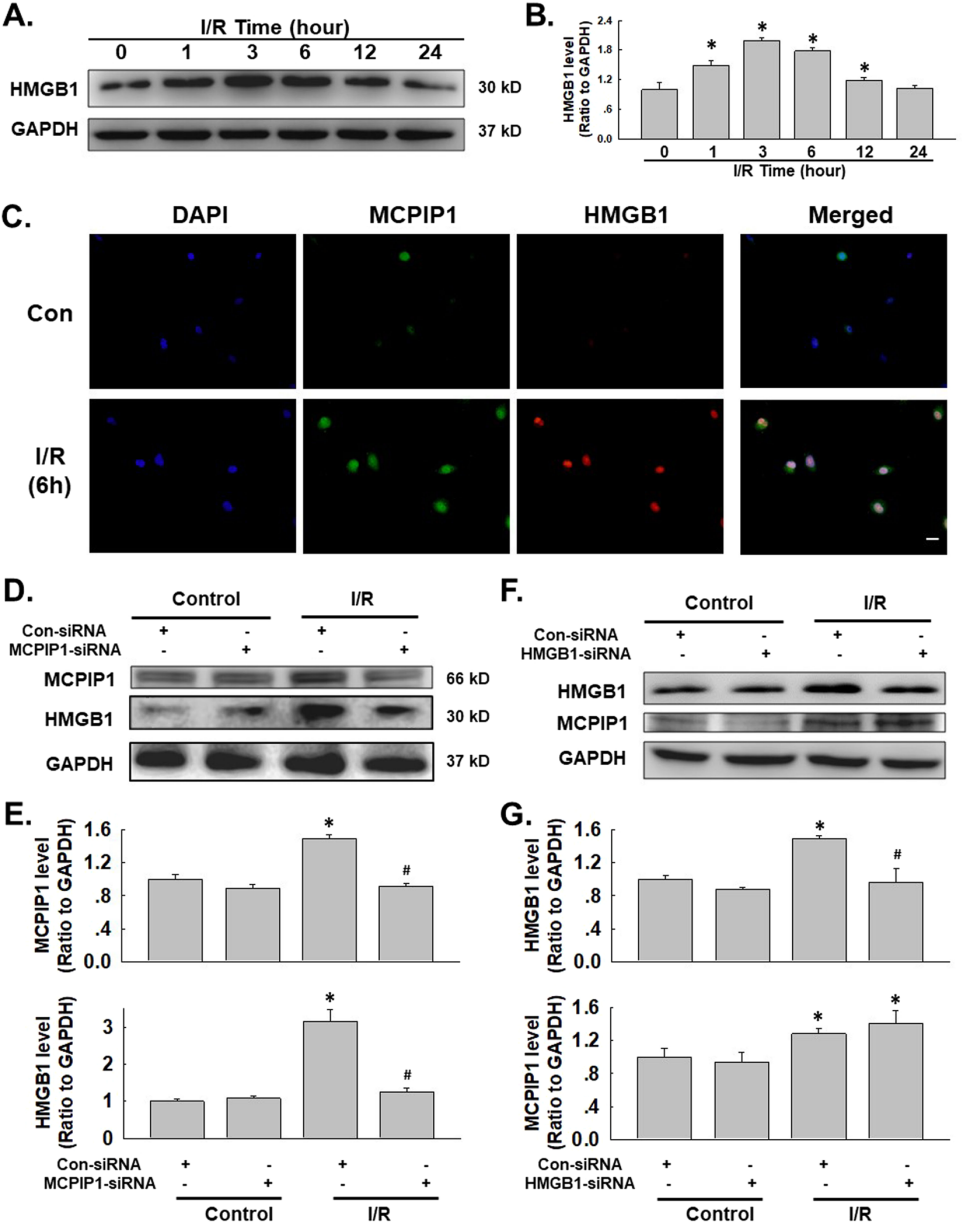
I/R induced HMGB1 expression in HUVECs. (**A**) Representative blots showing that I/R induced HMGB1 expression in a time-dependent manner. (**B**) Densitometric analyses of HMGB1 levels from four independent experiments; *p < 0.05 compared with the 0 h group. (**C**) Representative images of immunocytochemical staining showing that I/R induced MCPIP1 and HMGB1 expression in HUVECs. Scale bar, 20 μm. (**D**) Representative blots showing the effects of MCPIP1 siRNA on I/R-induced MCPIP1 and HMGB1 expression. (**E**) Densitometric analyses of MCPIP1 and HMGB1 levels from four independent experiments; *p < 0.05 compared with the control group. (**F**) Representative blots showing the effects of HMGB1 siRNA on I/R-induced MCPIP1 and HMGB1 expression. (**G**) Densitometric analyses of MCPIP1 and HMGB1 levels from four independent experiments; *p < 0.05 compared with the control group, ^#^p < 0.05 compared with the I/R group.

**Figure 2:**
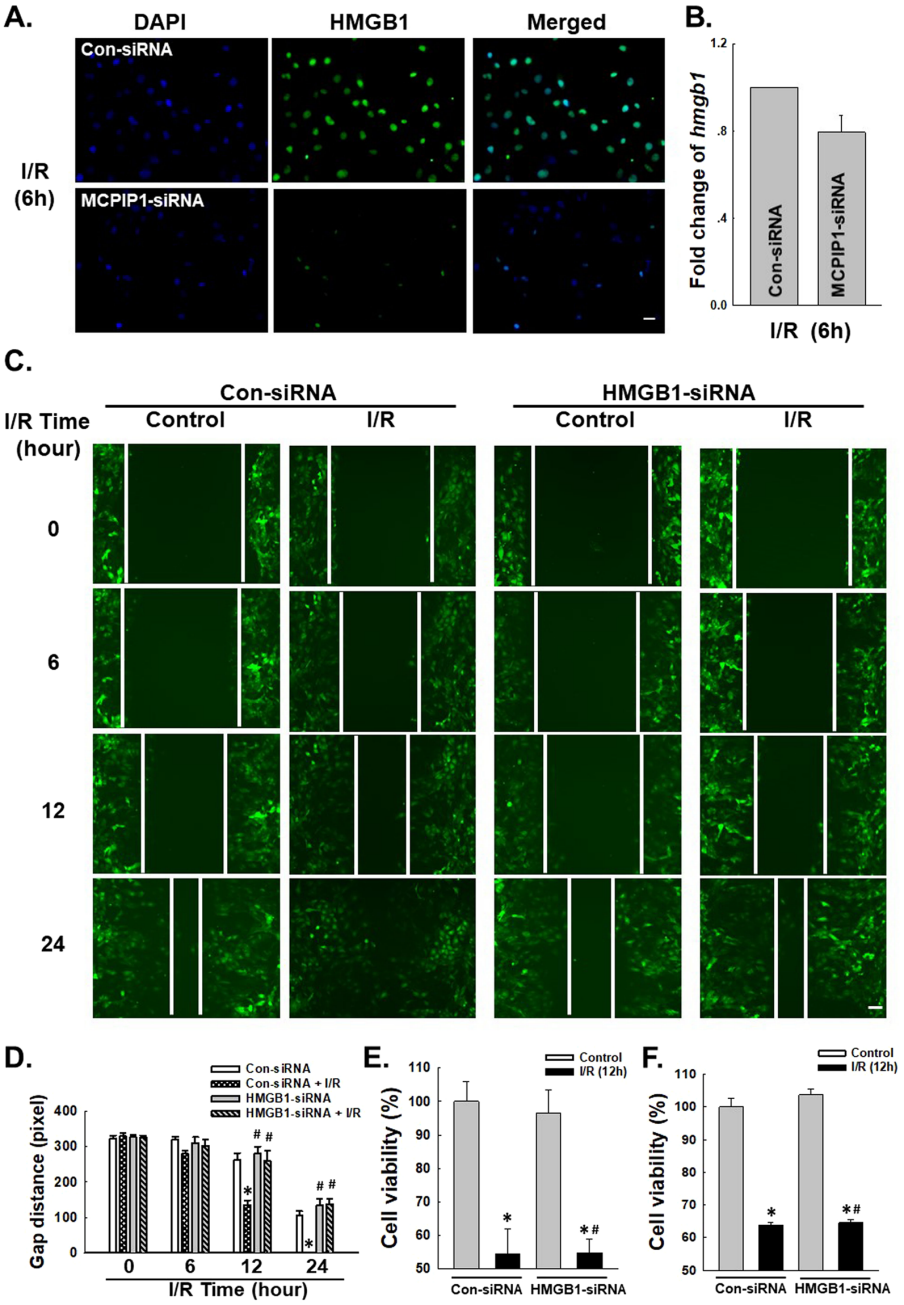
HMGB1 is involved in I/R-induced HUVEC migration but not apoptosis. (**A**) Representative images of immunocytochemical staining showing that I/R-induced HMGB1 expression was attenuated by MCPIP1 siRNA in HUVECs. Scale bar, 20 μm. (**B**) Results from quantitative RT-PCR demonstrating that the expression of *hmgb1* mRNA was not affected by MCPIP1 siRNA treatment in HUVEC cells, n = 3. (**C**) Representative images showing that the I/R-induced migration of HUVECs grown in monolayer cultures was abolished by HMGB1 siRNA. Scale bar, 80 μm. (**D**) The quantification of the scratch gap distance in six independent experiments is presented. *p < 0.05 compared with the control group. (**E**) MTT assay showing the effect of HMGB1 siRNA on cell viability following I/R treatment. *p < 0.05 compared with the control group, ^#^p < 0.05 compared with the I/R group. (**F**) CCK8 assay showing the effect of HMGB1 siRNA on cell viability following I/R treatment. *p < 0.05 compared with the control group, ^#^p < 0.05 compared with the I/R group.

Based on previous data from our lab, MCPIP1 expression is induced by I/R in HUVECs and is associated with cell migration and apoptosis, but the detailed mechanism remains unclear^1^. Interestingly, a recently study suggested a link between MCPIP1 and HMGB1 in macrophage activation/apoptosis and migration, indicating a role for HMGB1 in I/R-induced endothelial cell dysfunction^37^. HMGB1 induces endothelial cells to increase the expression of adhesion molecules^38^ and causes dendritic cell maturation^39^ through a downstream signaling pathway, such as the mitogen-activated protein kinase (MAPK) pathway or the nuclear factor kappa B (NF-κB) pathway, thereby facilitating cellular responses^40^. Therefore, we first examined the role of HMGB1 in the functional changes observed in HUVECs subjected to I/R. As shown in Fig. 1A,B, the exposure of HUVECs to I/R resulted in significant increases in the levels of the HMGB1 protein in a time-dependent manner, which was confirmed using immunofluorescence staining (Fig. 1C). The expression of both HMGB1 and MCPIP1 was down-regulated with siRNAs to investigate the association between up-regulated HMGB1 and MCPIP1 expression. As shown in Fig. 1D,E, MCPIP1 siRNA significantly inhibited the I/R-induced increase in MCPIP1 expression as expected; the I/R-induced increase in HMGB1 levels was also attenuated. Moreover, HMGB1 siRNA significantly inhibited the I/R-induced increase in HMGB1 expression, but not MCPIP1 expression, indicating that HMGB1 is downstream of MCPIP1 (Fig. 1F,G). The effect of MCPIP1 siRNA on HMGB1 levels was confirmed by immunocytochemistry staining (Fig. 2A). To further understand the mechanism by which MCPIP1 induced HMGB1 expression in I/R, the effect of MCPIP1 siRNA on *hmbg1* mRNA expression was measured. Interestingly, MCPIP1 siRNA had no significant effect on *hmbg1* mRNA expression (Fig. 2B), indicating that a post-transcriptional mechanism may be involved.

### HMGB1 is involved in I/R-mediated HUVEC migration but not apoptosis

As a feature of ischemic heart disease, abnormal angiogenesis is an important aspect of endothelial cell dysfunction, which is is characterized by migratory and proliferative phenotypes and the differentiation of endothelial cells into an angiogenic phenotype^1,4,8,41,42^. Apoptosis of endothelial cells is the initial step in angiogenesis and the regression of neo-vessels^1,3,11–13^. MCPIP1 has recently been implicated in the migration and apoptosis of HUVECs in response to I/R and several other stimuli^1,35^. Functional assays of cell migration and apoptosis were conducted to determine how up-regulated HMGB1 expression is associated with the I/R-induced increase in MCPIP1 expression. As shown in Fig. 2C,D, the results of the scratch assay revealed an increase in HUVEC migration following I/R, which was attenuated by HMGB1 siRNA. Interestingly, although HMGB1 is involved in cell apoptosis under different conditions, the HMGB1 siRNA did not alter the I/R-induced death of HUVECs (Fig. 2E,F), indicating that HMGB1 has a specific role in I/R injury.

### The effect of HMGB1 on HUVEC migration is mediated by autophagy

**Figure 3:**
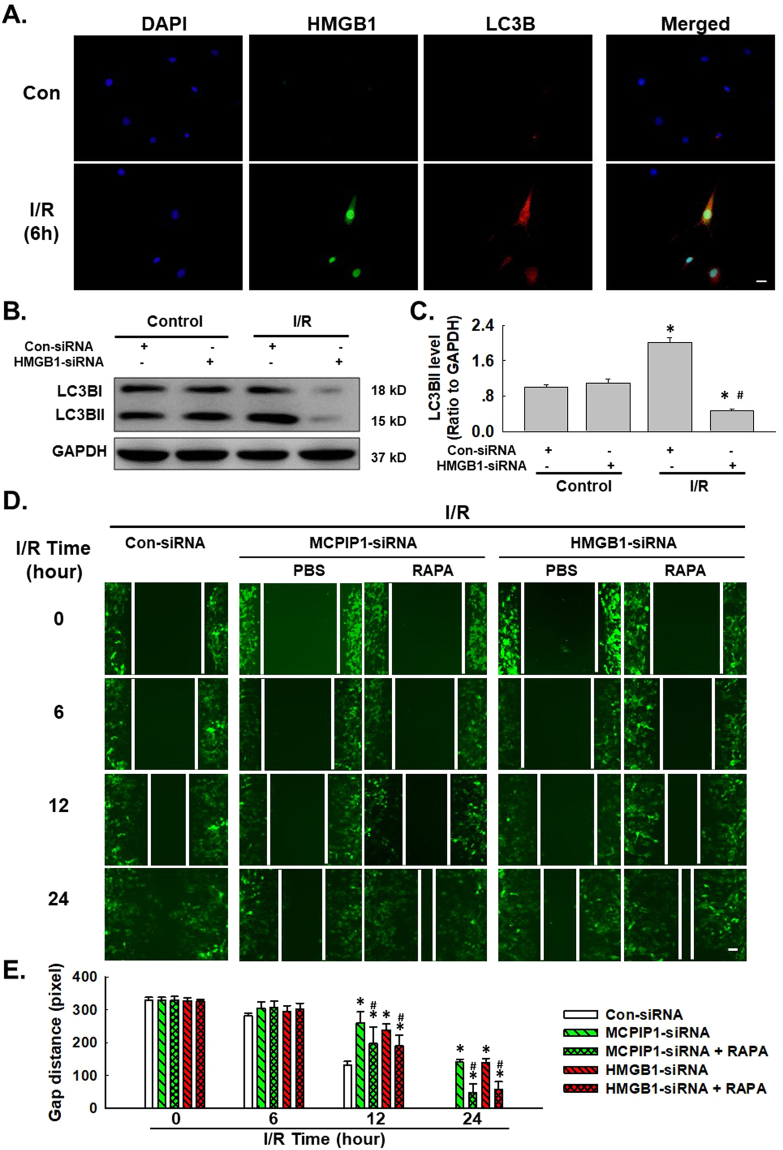
Autophagy mediated the effect of HMGB1 on I/R-induced cell migration. (**A**) Representative images of immunocytochemical staining showing that HMGB1 co-localized with the autophagy marker LC3B in HUVECs in response to I/R injury. Scale bar, 20 μm. (**B**) Representative blots showing that HMGB1 siRNA attenuated the I/R-induced increase in LC3B expression. (**C**) Densitometric analyses of LC3B levels from four independent experiments; *p < 0.05 compared with the control group, ^#^p < 0.05 compared with the I/R group. (**D**) Representative images depicting that the inhibitory effects of HMGB1 and MCPIP1 siRNAs on I/R-induced cell migration were reversed by pretreatment with rapamycin (1 μmol/L, 1 hour). Scale bar, 80 μm. (**E**) The quantification of the scratch gap distance in six independent experiments is presented. *p < 0.05 compared with the corresponding time point in the control group, ^#^p < 0.05 compared with the corresponding time point in the I/R group.

Previous data from our laboratory have suggested a role for autophagy in I/R-induced endothelial cell dysfunction^3^, whereas HMGB1 has recently been shown to play a role in autophagy^43^. However, researchers have not determined whether HMGB1-induced cell migration is related to autophagy. We first detected the level of the HMGB1 protein and the autophagy marker LC3B in HUVECs using immunofluorescence staining. HMGB1 and LC3B were co-localized in HUVECs after 6 h of exposure to I/R. Furthermore, HMGB1 siRNA significantly attenuated the I/R-induced increase in LC3B levels (Fig. 3B,C). HMGB1/MCPIP1 siRNAs and the autophagy activator rapamycin (RAPA) were used to investigate the role of autophagy in HMGB1-mediated migration. As shown in Fig. 3D,E, both the HMGB1 and MCPIP1 siRNAs attenuated I/R-induced cell migration, which was restored by pretreatment with RAPA (1 μmol/L) for 1 h. Therefore, MCPIP1 and HMGB1 induced cell migration by promoting autophagy in response to I/R injury.

### I/R-induced MCPIP1 expression is associated with increased CaSR expression in HUVECs

**Figure 4:**
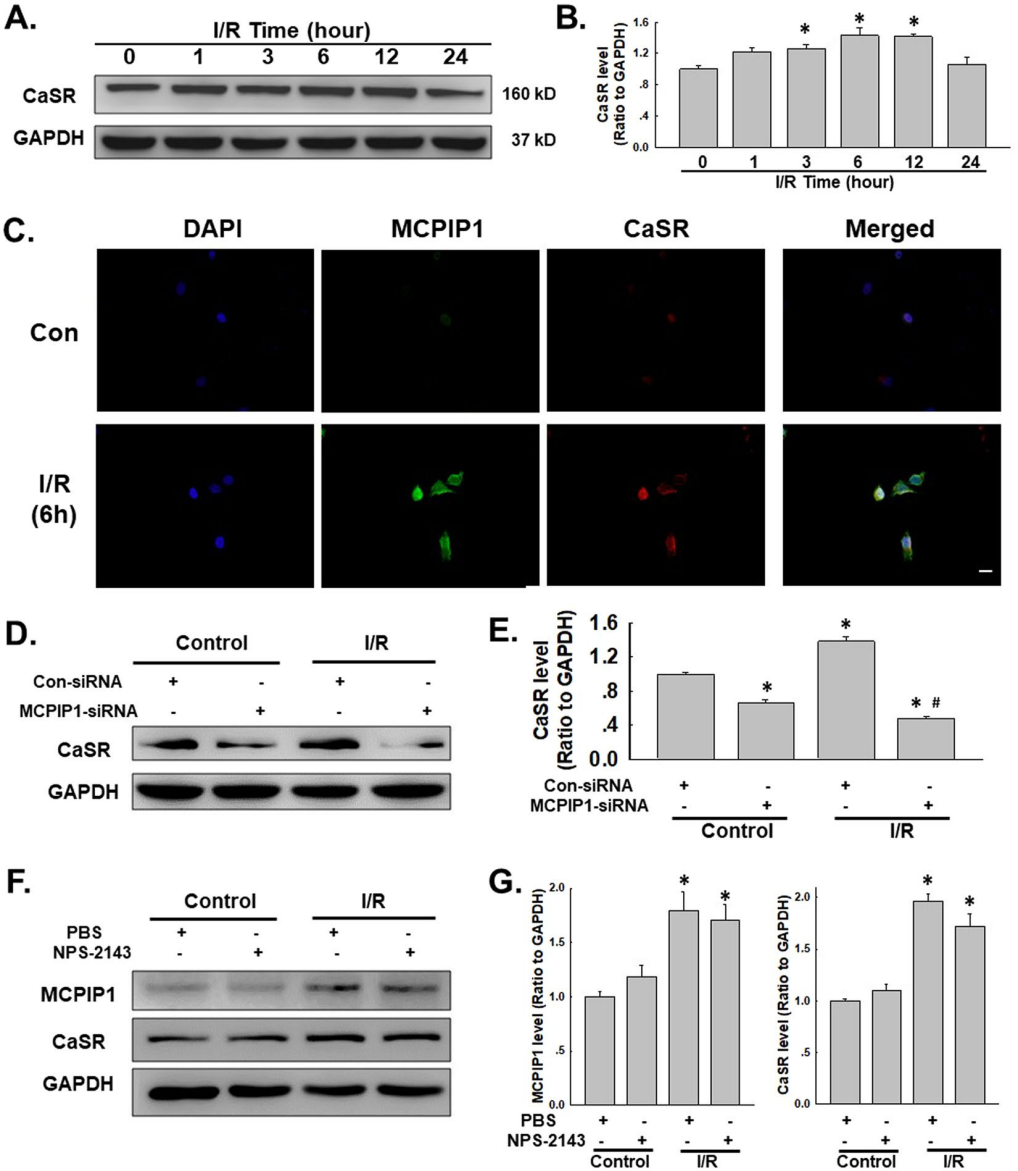
I/R induced CaSR expression in HUVECs. (**A**) Representative blots showing that I/R induced CaSR expression in a time-dependent manner. (**B**) Densitometric analyses of CaSR levels from four independent experiments; *p < 0.05 compared with the 0 h group. (**C**) Representative images of immunocytochemical staining showing that I/R induced MCPIP1 and CaSR expression in HUVECs. Scale bar, 20 μm. (**D**) Representative blots showing the effects of MCPIP1 siRNA on I/R-induced CaSR expression. (**E**) Densitometric analyses of CaSR expression from four independent experiments; *p < 0.05 compared with the control group. (**F**) Representative blots showing the effects of the CaSR-specific inhibitor NPS-2143 (5 μmol/L, 1 h) on I/R-induced MCPIP1 and CaSR expression. (**G**) Densitometric analyses of MCPIP1 and CaSR levels from four independent experiments; *p < 0.05 compared with the control group, ^#^p < 0.05 compared with the I/R group.

**Figure 5:**
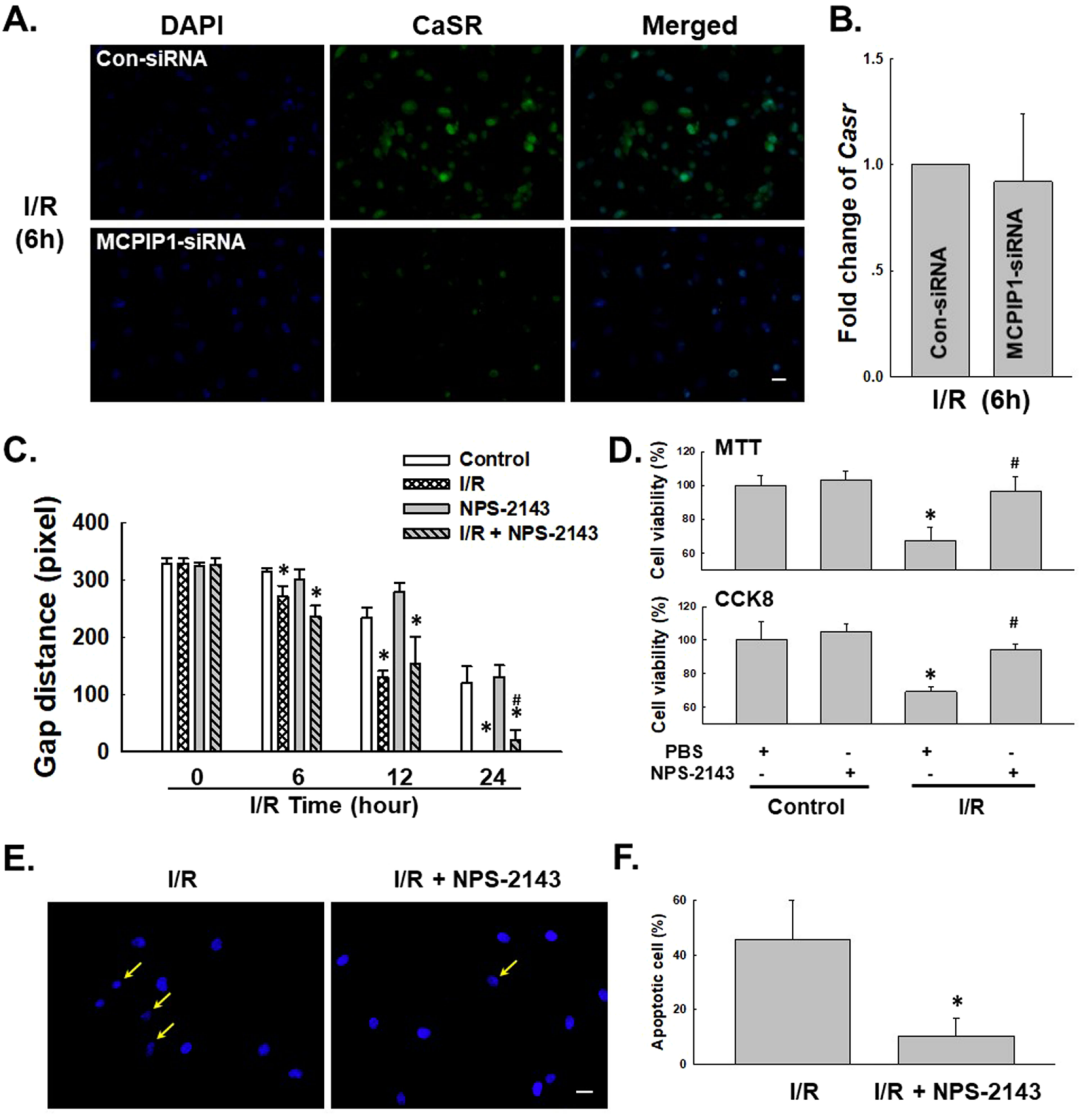
CaSR was involved in I/R-induced HUVEC cell apoptosis, but not migration. (**A**) Representative images of immunocytochemical staining showing that I/R-induced CaSR expression was attenuated by MCPIP1 siRNA in HUVECs. Scale bar, 20 μm. (**B**) Results from quantitative RT-PCR demonstrating that the expression of *casr* mRNA was not affected by MCPIP1 siRNA treatment in HUVEC cells, n = 3. (**C**) The quantification of the scratch gap distance in six independent experiments shows the effect of NPS-2143 on I/R-induced cell migration in HUVECs grown in monolayer cultures. *p < 0.05 compared with the corresponding time point in the control group, ^#^p < 0.05 compared with the corresponding time point in the I/R group. (**D**) MTT assay showing that NPS-2143 rescued cells from I/R-induced apoptosis. *p < 0.05 compared with the control group, ^#^p < 0.05 compared with the I/R group. (**E**) Representative images of Hoechst 33342 staining showing that I/R-induced HUVEC apoptosis was attenuated by NPS-2143 pretreatment. Scale bar, 10 μm. (**F**) Percentages of apoptotic cells from six independent experiments are presented. *p < 0.05 compared with the I/R group.

Previous data from our laboratory have suggested an important role for CaSR in I/R-induced cardiomyocyte death^27,44^. As HMGB1 was not involved in apoptosis, we next determined whether CaSR mediated HUVEC apoptosis. First, the CaSR level was detected in HUVECs exposed to the I/R model. As shown in Fig. 4A,B, I/R induced CaSR expression in a time-dependent manner, with a peak at 6 h of reperfusion, which was confirmed by immunofluorescence staining (Fig. 4C). MCPIP1 expression in HUVECs was down-regulated with an siRNA to further examine the link between MCPIP1 and CaSR. As shown in Fig. 4C,D, MCPIP1 siRNA not only significantly inhibited the I/R-induced increase in CaSR expression but also decreased the basal CaSR levels in HUVECs, indicating a strong relationship between MCPIP1 and CaSR. The CaSR-specific inhibitor NPS-2143 was used to clarify the relationship between MCPIP1 and CaSR^45^. As shown in Fig. 4D,E, inhibition of CaSR with NPS-2143 (5 μmol/L) had no effect on MCPIP1 and CaSR expression. Based on the results shown in Fig. 4C–E, the I/R-induced increase in CaSR expression occurred downstream of MCPIP1 signaling in HUVECs. The effect of MCPIP1 siRNA on CaSR levels was confirmed by immunocytochemistry (Fig. 5A). To further understand the mechanism by which MCPIP1 induced HMGB1 expression in I/R, the effect of MCPIP1 siRNA on *casr* mRNA expression was measured. Interestingly, MCPIP1 siRNA had no significant effect on *casr* mRNA expression (Fig. 5B), indicating that a post-transcriptional mechanism may be involved.

### CaSR is involved in I/R-mediated HUVEC apoptosis but not migration

NPS-2143 was used to investigate the role of CaSR in I/R-induced cell apoptosis and migration to further examine the functional role of CaSR in HUVECs. As shown in Fig. 5C, NPS-2143 alone did not alter HUVEC migration, and the I/R-induced increase in migration was not prevented by pretreatment with NPS-2143, excluding the possibility that CaSR participates in I/R-induced endothelial cell migration. Interestingly, although NPS-2143 pretreatment alone did not induce cell proliferation, this pretreatment rescued HUVECs from I/R-induced death as determined using MTT and CCK8 assays (Fig. 5D,E). This finding was confirmed by staining the cells with Hoechst 33342, a dye that specifically stains nuclei^1^. As shown in Fig. 5F,G, condensation and fragmentation of nuclei, which are characteristic features of apoptotic cells, were observed in HUVECs that had been subjected to I/R for 6 h. The I/R-induced cell death was significantly ameliorated by pretreatment with NPS-2143, as indicated by the appearance of normal cells characterized by regular and round nuclei (Fig. 5F,G).

### The effect of CaSR on HUVEC apoptosis is mediated by autophagy

**Figure 6:**
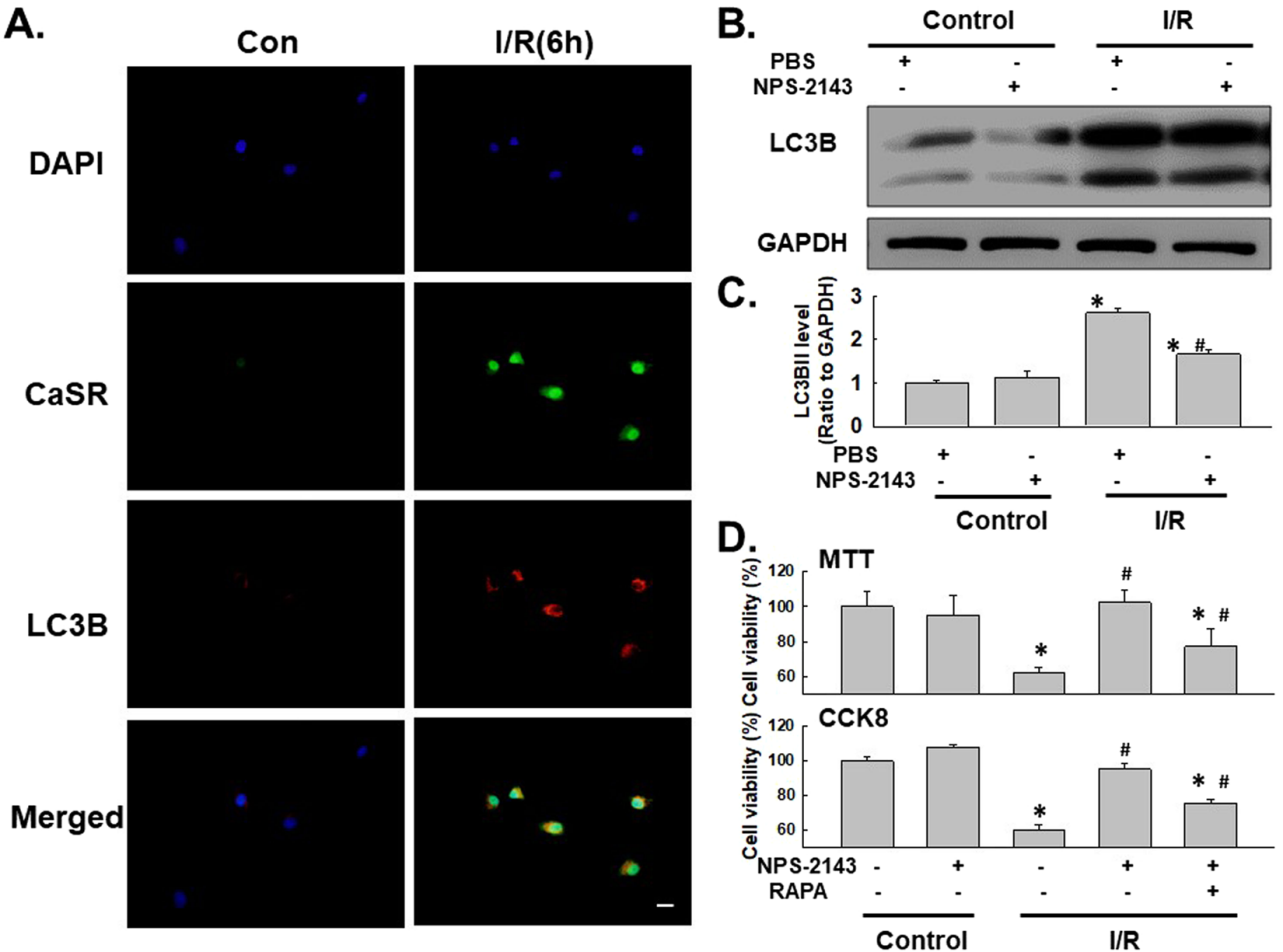
Autophagy mediated the effect of CaSR on I/R-induced cell apoptosis. (**A**) Representative images of immunocytochemical staining showing that CaSR co-localized with the autophagy marker LC3B in HUVECs following I/R injury. The I/R-induced increase in the level of LC3B, but not CaSR, was attenuated by NPS-2143 pretreatment. Scale bar, 20 μm. (**B**) Representative blots showing that NPS-2143 pretreatment attenuated the I/R-induced increase in LC3B expression. (**C**) Densitometric analyses of LC3B expression from four independent experiments; *p < 0.05 compared with the control group, ^#^p < 0.05 compared with the I/R group. (**D**) MTT assay showing that the rescue effect of NPS-2143 on HUVECs subjected to I/R injury was reversed by treatment with rapamycin. n = 6, *p < 0.05 compared with the control group, ^#^p < 0.05 compared with the I/R group.

Recent studies have suggested a link between CaSR and autophagy under different conditions^46,47^. Although the role of autophagy in the apoptosis of HUVECs subjected to I/R injury has been determined, researchers have not determined whether the effect of CaSR on I/R-induced cell death is mediated by autophagy. The levels of the CaSR protein and the autophagy marker LC3B in HUVECs were detected using immunofluorescence staining. CaSR and LC3B were co-localized in HUVECs exposed to 6 h of I/R. Moreover, NPS-2143 pretreatment attenuated the I/R-induced increase in LC3B expression in HUVECs, indicating a direct link between CaSR and autophagy (Fig. 6A), which was confirmed by western blotting (Fig. 6B,C). HUVECs were treated with both NPS-2143 and RAPA and analyzed using the MTT assay to further examine the functional relationship between CaSR and autophagy. As shown in Fig. 6D, NPS-2143-mediated inhibition of cell death was reversed by RAPA treatment, indicating that the effect of CaSR on I/R-induced cell death was mediated by autophagy.

## Discussion

Whereas many studies have examined I/R injury in cardiomyocytes, the direct effect of I/R on endothelial cells has received less attention. Apoptosis occurs in endothelial cells in the very early stages of reperfusion following the radial spread of apoptosis to surrounding cardiac myocytes, indicating that soluble pro-apoptotic mediators are released from endothelial cells to promote myocyte death^48^. Cytokines, chemokines and oxidative stress have been shown to be involved in this process^1,3,49^. The aim of the current study was to investigate the downstream events mediated by endothelial cell-derived MCPIP1 that lead to cell activation and migration in response to I/R injury.

MCPIP1 plays an important role in inflammatory diseases, such as pneumoconiosis^16^, neuronal injury^50^, and I/R injury^1^. MCPIP1 expression is rapidly increased in HUVECs after I/R, whereas most functional changes occur several hours after reperfusion; therefore, the downstream pathway is of interest. Recently, studies have identified a link between MCPIP1 and HMGB1 under various conditions^37,50^, indicating a potential role for the interaction between MCPIP1 and HMGB1 in I/R-induced endothelial cell dysfunction. HMGB1 has been reported to exert multiple cardioprotective effects^51^. Interestingly, although HMGB1 mediated I/R-induced cell migration, which is consistent with previous studies of HMGB1 and cell migration^52–54^, HMGB1 did not have an effect on cell viability. However, contradictory reports have been published regarding the role of HMGB1 in apoptosis in different cell types. HMGB1 exerts a protective effect on cardiomyocytes, but it also induces endothelial progenitor cell apoptosis through the receptor for the advanced glycation end products (RAGE)-dependent protein kinase R-like ER kinase (PERK)/eukaryotic translation initiation factor 2 alpha (eIF2α) pathway^51,55^, indicating that HMGB1 has a complex role in cell apoptosis. HMGB1 is associated with autophagy under various conditions^43^, and our results provided another example of an association between HMGB1 and autophagy.

Apoptosis is an important aspect of endothelial cell dysfunction. Although CaSR is involved in I/R-induced cardiomyocyte death, further studies are required to determine whether the I/R-induced increase in CaSR expression is a general phenomenon. CaSR not only responds to extracellular Ca^2+^ but is also activated by many ligands, such as divalent and trivalent cations, L-amino acids, and polyamines^56^. Although most reports indicate a role for CaSR in apoptosis, it also promotes osteoblast proliferation during bone remodeling^57^. In the current study, NPS-2143, a CaSR-specific inhibitor^45^, rescued cells stimulated by I/R from apoptosis and increased the levels of LC3B, a marker of autophagy, clearly indicting that CaSR plays a role in apoptosis associated with endothelial cell dysfunction. Furthermore, the preventative effect of NPS-2143 was reversed by treatment with rapamycin, a specific autophagy activator, strongly indicating that CaSR induced apoptosis via autophagy. Interestingly, CaSR was involved in cell apoptosis, but it had no effect on cell migration, suggesting that HMGB1 and CaSR coordinately regulated I/R-induced endothelial cell dysfunction. Moreover, both HMGB1 and CaSR affect downstream functions via autophagy, suggesting that autophagy has a key role in endothelial cell dysfunction, which is consistent with our previous findings of autophagy in endothelial cells subjected to I/R injury^3^. Interestingly, MCPIP1 siRNA did not affect *hmgb1* or *casr* mRNA expression, indicating that a post-transcriptional mechanism may be involved. In fact, a recent study from our group found that circRNA was involved in the effect of MCPIP1/ZC3H12A on downstream cascades via ubiquitination. Whether MCPIP1 also regulates HMGB1 and CaSR through a post-transcriptional mechanism warrants further investigation.

**Figure 7:**
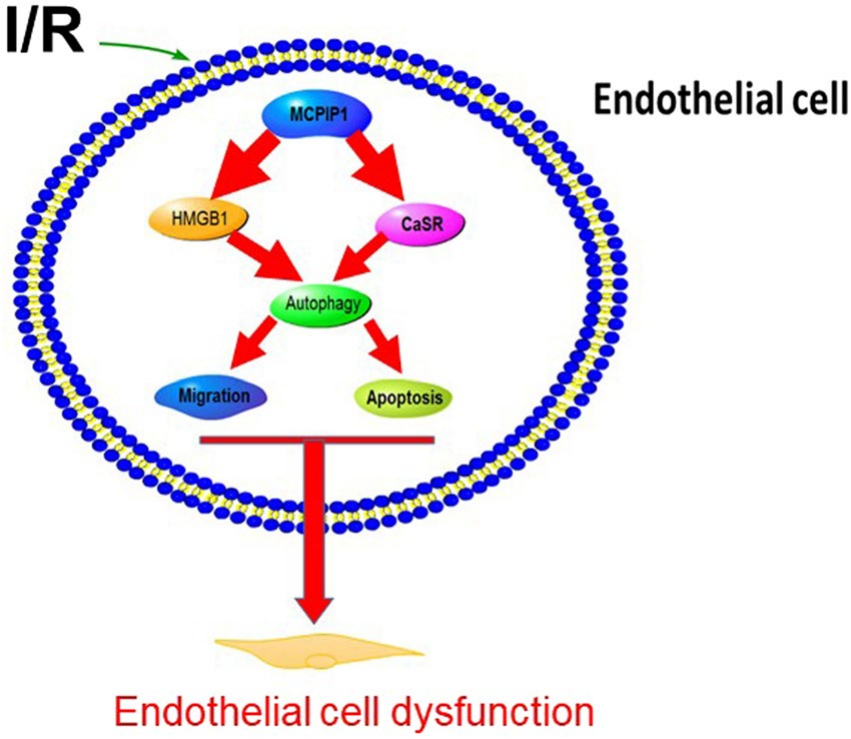
Schematic model of the mechanism by which HMGB1, CaSR, and autophagy promote I/R-induced endothelial cell dysfunction.

In summary, our study identified a mechanism by which MCPIP1 regulates endothelial cells via HMGB1 and CaSR in response to I/R. Furthermore, I/R-mediated both HMGB1 and CaSR expression affected cell migration and apoptosis via autophagy, resulting in upregulation of angiogenesis and apoptosis during the late stages of I/R injury (Fig. 7). These findings have implications for I/R injury in heart failure patients. A better understanding of the mechanisms regulating MCPIP1 and autophagy may aid in the development of therapeutic strategies to treat I/R injuries.

## Electronic supplementary material

Supplementary data
