# Supplementary material for: MCPIP1-induced autophagy mediates ischemia/reperfusion injury in endothelial cells via HMGB1 and CaSR
Source: Sci Rep. 2018 Jan 29;8:1735. doi: 10.1038/s41598-018-20195-6 (PMC5788920; doi:10.1038/s41598-018-20195-6)
Supplement: Supplementary file 1 — Supplementary data [file 41598_2018_20195_MOESM1_ESM.doc]

**Supplementary information**

**Manuscript title: MCPIP1-induced autophagy mediates ischemia/reperfusion injury in endothelial cells via HMGB1 and CaSR**

Authors: Xiaolong Xie, Tiebing Zhu, Lulu Chen, Shuang Ding, Han Chu, Jing Wang, Honghong Yao, and Jie Chao

**Table of contents**

Supplementary Figure S1 1

**Supplementary Figure S1**


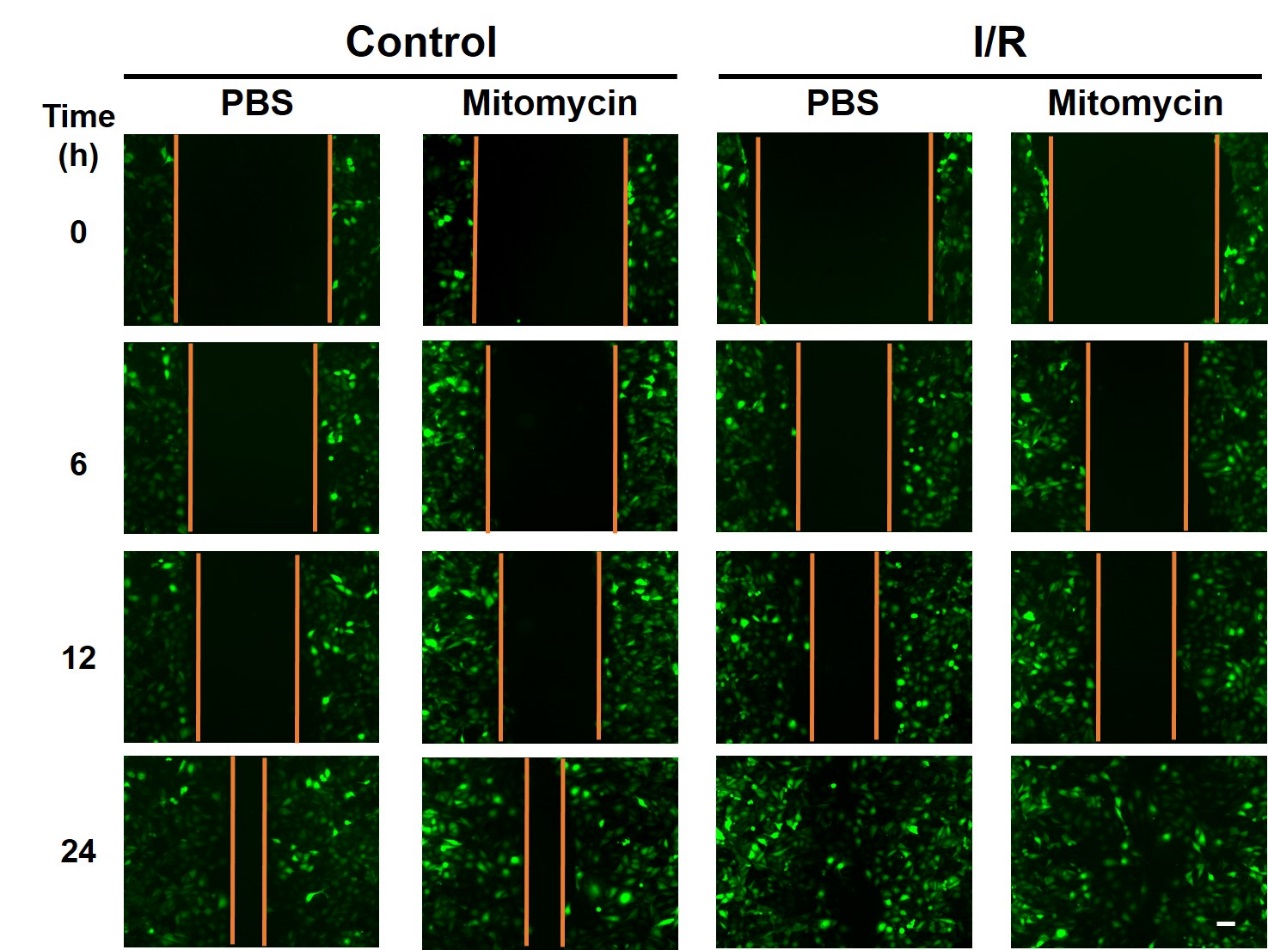


**Figure S1 Effect of mitomycin on cell migration.**

Representative images showing that the I/R-induced migration of HUVECs in monolayer cultures was not altered by 1 hour of pretreatment with mitomycin. Scale bar, 80 μm.
